# Effect of temperature on the biological parameters of the cabbage aphid *Brevicoryne brassicae*

**DOI:** 10.1002/ece3.4639

**Published:** 2018-11-11

**Authors:** Bernard Steve Baleba Soh, Sevilor Kekeunou, Samuel Nanga Nanga, Michel Dongmo, Hannah Rachid

**Affiliations:** ^1^ International Institute of Tropical Agriculture Yaoundé Cameroon; ^2^ Laboratory of Zoology Faculty of Science University of Yaoundé 1 Yaoundé Cameroon

**Keywords:** *Brevicoryne brassicae*, life table, phenological models, temperature

## Abstract

The cabbage aphid, *Brevicoryne brassicae,* is a pest of many plants of the Brassicaceae family including cabbage, *Brassica oleracea* Linnaeus, 1753. We investigated the effect of temperature on the biological parameters of *B. brassicae* using different temperature‐based models incorporated in the Insect Life Cycle Modelling (ILCYM) software. Nymphs of first stage were individually placed in the incubators successively set at 10°C, 15°C, 20°C, 25°C, 30°C, and 35°C; 75 ± 5% RH; and L12: D12‐hr photoperiods. We found that first nymph reached the adult stage after 18.45 ± 0.04 days (10°C), 10.37 ± 0.26 days (15°C), 6.42 ± 0.07 days (20°C), 5.076 ± 0.09 days (25°C), and 5.05 ± 0.10 days (30°C), and failed at 35°C. The lower lethal temperatures for *B. brassicae* were 1.64°C, 1.57°C, 1.56°C, and 1.62°C with a thermal constant for development of 0.88, 0.87, and 0.08, 0.79 degree/day for nymphs I, II, III, and IV, respectively. The temperatures 10, 30, and 35°C were more lethal than 15, 20, and 25°C. Longevity was highest at 10°C (35.07 ± 1.38 days). Fertility was nil at 30°C and highest at 20°C (46.36 ± 1.73 nymphs/female). The stochastic simulation of the models obtained from the precedent biological parameters revealed that the life table parameters of *B. brassicae* were affected by the temperature. The net reproduction rate was highest at 20°C and lowest at 30°C. The average generation time decreased from 36.85 ± 1.5 days (15°C) to 6.86 ± 0.1 days (30°C); the intrinsic rate of increase and the finite rate of increase were highest at 25°C. In general, the life cycle data and mathematical functions obtained in this study clearly illustrate the effect of temperature on the biology of *B. brassicae*. This knowledge will contribute to predicting the changes that may occur in a population of *B. Brassiace* in response to temperature variation.

## 1 INTRODUCTION

The cabbage aphid, *Brevicoryne brassicae* Linnaeus, 1758 (Hemiptera: Aphididae), is native to Europe (Kessing & Mau, 1991), but now, it is distributed throughout the temperate and warm temperate zones of the world (Blackman & Eastop, 2000). This aphid is a specialist of the plants of the Brassicaceae family (Gabrys et al., 1997; Pontoppidan, Hopkins, Rask, & Meijer, 2003), and it is a significant pest of cabbage (Fathipour, Hosseini, Talebi, & Moharramipour, 2006). By forming colonies on the stems, petioles, and leaves of their hosts, *B. brassicae* causes different damages that Christelle (2007) and Eaton (2009) grouped into two categories: (a) direct damage due to the host sap absorption; and (b) indirect damage: associated with the transmission phytopathogenic viruses (Blackman & Eastop, 2000; Chan, Forbes, & Raworth, 1991) and the ejection of honeydew causing fumagine formation that affects cabbage photosynthetic activity. Under severe infestation, *B. brassicae* reduces the production of cabbage from 70% to 80% (Khattak, Hameed, Khan, & Farid., 2002; Rustamani, Qamikhani, Munshi, & Chutto, 1988).

Aphids are ectothermic organisms; all their physiological processes largely depend on several climatic variables that include temperature (Brodeur et al., 2013). According to Campbell, Frazer, Gilbert, Gutierrez, and Mackauer (1974), the temperature is a critical abiotic factor affecting insect biology. The rise in temperature from 1.5 to 5.8°C until the end of 2,100 as predicted by many mathematical models (Govindasamy, Duffy, & Coquard, 2003; Hijmans, Cameron, Parra, Jones, & Jarvis, 2005; IPCC, 2014) is likely to increase the metabolic activity of *B. brassicae*. The ability of insects to modify their physiology and behavior in response to an environmental factor is termed phenotypic plasticity (Pigliucci, Murren, & Schlichting, 2006). This plasticity is controlled by several physiological mechanisms (transcription, translation, enzyme, and hormonal regulation) that produce local or systemic responses (Whitman & Agrawal, 2009). These responses can be visualized using mathematical functions called reaction norms, which plot values for a specific phenotypic trait across two or more environments or treatments (David et al., 1997). According to Fischer and Karl (2010), phenotypic plasticity is a powerful and effective mechanism used by different organisms to cope with the detrimental effects of short‐term environmental changes. In the context of global warming, the primary challenge faced by ecologists is to predict variation that occurs on the biology of ectotherm organisms (Brodeur et al., 2013). Therefore, temperature‐based reaction norms are essential analytical tools for evaluating, understanding, and predicting the phenotypic variation in insects (Baker, 1991; Jarvis & Baker, 2001).

There are two distinct modeling approaches (Kroschel et al., 2013; Trnka et al., 2007). (a) The first approach is the inductive method, which matches the climate where an organism is usually found within a region to where it is not found using long‐term meteorological data (Beaumont, Hughes, & Poulsen, 2005; Legaspi & Legaspi, 2007; Peacock & Worner, 2006; Sutherst & Maywald, 2005; Trnka et al., 2007). This modeling approach has an advantage to only depend on the presence/absence data of the species studied. However, the critical limitation of this approach is its failure to consider the biological characteristics of the species in the modeling framework (Venette et al., 2010). (b) The second approach is deductive, which use mathematical functions (process‐based climatic response) to describe the basic physiological principles of the insect species growth, namely its development, survival, and reproduction (Curry, Feldman, & Smith, 1978; Nietschke, Magarey, Borchert, Calvin, & Jones, 2007; Sporleder, Kroschel, Quispe, & Lagnaoui, 2004; Trnka et al., 2007). This approach is based on detailed laboratory experiments that produce life table parameters and allows the simulation of populations according to real or interpolated data for a given region and time (Sporleder, Simon, Juarez, & Kroschel, 2008).

Modeling process uses linear and nonlinear models. Linear models have long been used for the construction of phenological patterns of insect populations (Roltsch, Mayse, & Clausen, 1990); they produce better predictions at intermediate temperatures. Due to the nonlinear nature of insect demographic parameters (development rate, mortality, fertility, etc.) at low and high temperatures, nonlinear models were developed to give accurate results at extreme temperatures (Briere, Pracros, Le Roux, & Pierre, 1999; Logan, Wollkind, Hoyt, & Tanigoshi, 1976; Sharpe & DeMichele, 1977). Their development requires knowledge of lethal temperatures (upper and lower) of insect species studied as well as data collected on each individual from birth to death (Nietschke et al., 2007). Several studies have already been conducted to evaluate the effect of temperature on the biology of *B. brassicae* (Abdel‐Rahman et al.; 2011; Akinlosotu, 1977; DeLoach, 1974; Fathipour, Hosseini, Talebi, Moharamipour, & Asgari, 2005; Gupta, 2014; Satar, Kerstng, & Ulusoy, 2005); however, these studies do not include reaction norms in their studies. Therefore, the main objective of this work was to study and visualize the effect of temperature on the biological parameters of *B. brassicae* using mathematical models found in the Insect Life Cycle Modelling (ILCYM) software (Tonnang et al., 2013). These models will serve as essential components to predict the distribution of *B. brassicae* as influenced by the temperature.

## 2 MATERIALS AND METHODS

### 2.1 Insect culture

The population of *Brevicoryne brassicae* used in this study was a clonal line of aphids (to avoid genetic divergence) collected initially in a single infested cabbage farm in Dschang (05° 26′70″N. 010° 04′09″E; Al. 1,391 m), a town situated in western Cameroon. The stock culture of aphids was cultured on the potted cabbage plants (*Brassica oleracea* var. Marcanta L) in an air‐controlled insectary room maintained at 25 ± 1°C, 75% ± 5% RH, and 12L:12D‐hr photoperiods. Aphids were reared in the insectary for 2–3 generations before individuals were harvested for the experiments (Kindlmann & Dixon, 1989). Cabbage plants used in the experiments were obtained from a greenhouse culture at International Institute of Tropical Agriculture (IITA)—Cameroon—situated in Nkolbisson, a peripheric west quarter of Yaounde (11°5′ N. 3°86′ E).

### 2.2 Experimental design

#### 2.2.1 Rearing conditions

The effect of temperature on the biological parameters of *Brevicoryne brassicae* was studied in cohorts of single life stages in controlled environmental chambers at six constant temperatures, that is, 10, 15, 20, 25, 30, and 35°C, of 75% ± 5% RH, and maintained on a photoperiod regime of 12L:12D‐hr photoperiods. The required temperatures and hygrometry inside the incubators were regularly monitored using a standard thermo‐hygrometer of HOBO trademark.

#### 2.2.2 Experimental conditions

**Figure 1:**
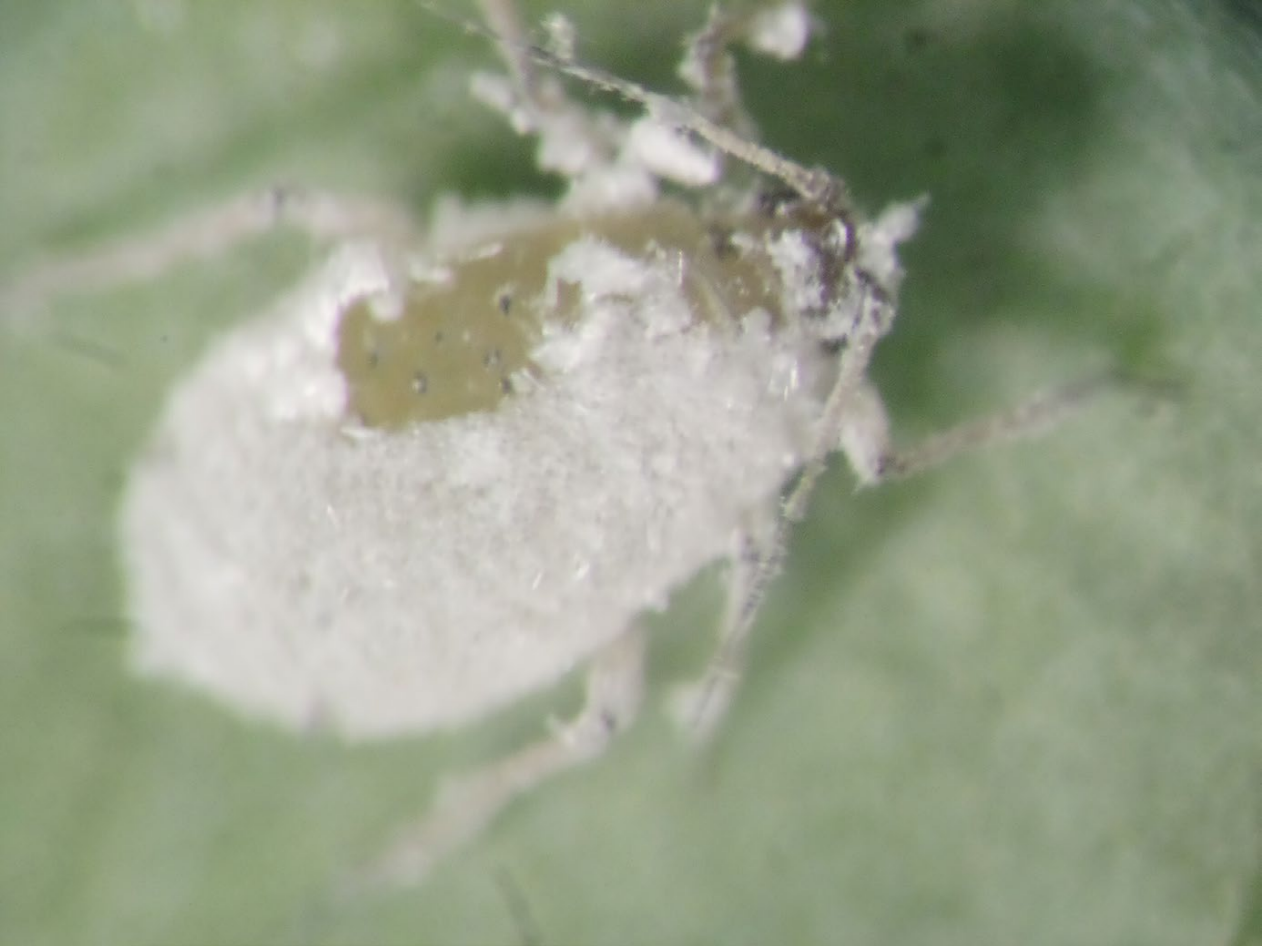
Cabbage aphids, *Brevicoryne brassicae*, Photo: Baleba Steve

One hundred cabbage leaves were received each two to three apterous individual of *Brevicoryne brassicae* from the stock culture (Figure 1). Leaves were kept hydrated by immersing their petioles in distilled water in a glass tube (12 ml) sealed with parafilm. Each cabbage leave with aphids was transferred inside a clear plastic container covered with a lid previously perforated at the center and closed with an organza tissue to aerate the container. After 24 hr, one newly born nymph was maintained on each cabbage leaf; adult and the extra nymphs were removed. After that, a total of 100 nymphs were individually monitored at each test temperature. Development and mortality of the different nymph stages were recorded daily. When they reached the adult stage, individuals were monitored daily to count and separate newborn nymphs from checking for the reproduction data. For each experimental temperature, aphid individuals were followed until the death. To avoid the effect of age on the survivorship and reproduction, aphids were carefully transferred on new cabbage leaves every 7 days.

### 2.3 Statistical analysis and modeling

#### 2.3.1 Software description

To study and visualize the effect of temperature on the biological parameters of *Brevicoryne brassicae,* we used the Insect Life Cycle Modelling (ILCYM, version 3.0) software developed by the International Potato Centre (CIP) and freely available at the CIP website: http://www.cipotato.org (Tonnang et al., 2013). Insect Life Cycle Modelling contains three modules: the model builder, the validation and simulation module, and the potential population distribution and risk mapping; we used the two‐first modules for the present study. ILCYM's model builder is a complete modeling interface that helps to develop insect temperature‐based models. It provides several nonlinear functions, which are adequate for describing the temperature dependency of the different processes in the species life history (i.e., development, survival, and reproduction). The Akaike's information criterion (AIC; Akaike, 1973), which is inbuilt in ILCYM, was used to select the best mathematical expression for each nymphal stage of *B. brassicae*. The validation and simulation module with the stochastic simulation tool was used to estimate life table parameters at constant temperatures based on the developed temperature‐based models.

#### 2.3.2 Distribution model of development times

For estimation of the variation among individuals in developmental times of *Brevicoryne brassicae,* the concepts of rate summation (Curry et al., 1978) and same shape (Sharpe, Curry, DeMichele, & Cole, 1977) were included. These concepts assumed that the intrinsic distributions of insect development times at different constant temperatures have the same shape (i.e., the distributions at different temperatures will fall on top of each other when “normalized” by a selected value such as the mean or median of each distribution). The data collected from different constant temperatures were fitted to three dichotomic models: logit, probit, and complementary log. Due to its lowest AIC value, the logit model represented by the following function was selected to depict the effect of temperature on the developmental time of the four nymphal stages of *B. brassicae*.

$$F\left(x\right)\;=\;\frac{1}{1\;+\;\exp\left(-\;(\text{ai}\;+\;\text{blnx})\right)}$$

where *F*(*x*) is the probability to complete development at time *x*, lnx is the natural logarithm of the days observed, *a* is the intercept corresponding to temperature *i*, and *b* is the common slope of the regression model.

#### 2.3.3 Temperature‐dependent development rate model

Development rate was expressed by the reciprocal of the mean development times for all the four nymphal stages of *Brevicoryne brassicae*. The relationship between temperature and development rate was described by the nonlinear Sharpe & DeMichele 1 model (Sharpe & DeMichele, 1977) for nymph I stage; while nymph II, III, and IV stages were described by the modified Janisch 1 model (Janisch, 1932). We chose the precedent models based on their respective AIC value that was smaller compared to those of other models; their mathematical equations are respectively expressed below:

$$r\left(T\right)\;=\;\frac{P\frac{T}{T_{0}}\exp[\frac{\Delta H_{A}}{R}(\frac{1}{T_{0}}\;-\;\frac{1}{T})]}{1\;+\;\exp[\frac{\Delta H_{L}}{R}(\frac{1}{T_{L}}\;-\;\frac{1}{T})]\;+\;\exp[\frac{\Delta H_{H}}{R}(\frac{1}{T_{H}}\;-\;\frac{1}{T})]}$$

$$r\left(T\right)\;=\;\frac{2}{D_{\min}\left[\text{expK}\left(T\;-\;T_{\mathrm{opt}}\right)\;+\;\text{exp}\;-\;K\left(T\;-\;T_{\mathrm{opt}}\right)\right]}$$

where *r*(*T*) is the development rate at temperature *T* (°K), *R* is the universal gas constant (1.987 cal/degree/mol), p represents the development rate at optimum temperature *T*_opt_ (°K) assuming no enzyme inactivation, Δ*H*_A_ is the enthalpy of activation of the reaction catalyzed by an enzyme (cal/mol/1), Δ*H*_L_ and Δ*H*_H_ are the change in enthalpy at high temperature (cal/mol/1), and *T*_H_ is the high temperature at which enzyme is half active. *T*_opt_ is the optimum temperature for survival (°C).

For each *B. brassicae* immature stage, the following linear regression equation was used to evaluate the lower developmental threshold (*T*_0_) and the thermal constant (*K*) expressed in degree days (DD)

r(T)=a+bT

where *r*(*T*) is the rate of development at temperature *T*,* a* is the y‐intercept, and *b* is the slope. The lower developmental threshold (*T*_0_) and the degree‐day (DD) requirement were estimated using the parameters: $T_{0}\;=\;-a/b$ and DD=1/b

#### 2.3.4 Temperature‐dependent mortality model

The mortality rate of each *Brevicoryne brassicae* immature stage was calculated by dividing the number of individuals that did not develop successfully to the next stage by the initial number of individuals at each stage. The effect of temperature on the mortality rate of *B. brassicae* was described by using polynomial 2 function for nymph I and II according to the equation below:

$$m\left(T\right)\;=\;\exp\left(a\;+\;bT\;+\;cT^{2}\right)$$

*m*(*T*) is the rate of mortality at temperature *T*(°C), and *a*,* b*,* c* are the equation parameters.

Wang 1 and 7 functions (Wang, Lan, & Ding, 1982) were used to illustrate the temperature dependence of mortality in nymphs III and IV, respectively. The following equations were used:

$$m\left(T\right)\;=\;1\;-\;\frac{1}{\exp\left[\left(1\;+\;\exp\left(-\frac{T\;-\;T_{\mathrm{opt}}}{B}\right)\right)\;+\;\left(1\;+\;\exp\left(-\frac{T_{\mathrm{opt}}\;-\;T}{B}\right)\right)\;\times \;H\right]}$$

$$m\left(T\right)\;=\;1\;-\;\frac{H}{\exp\left[1\;+\;\exp\left(-\frac{T\;-\;T_{\mathrm{opt}}}{B1}\right)\left(1\;+\;\exp\;-\;\frac{T_{\mathrm{opt}}\;-\;T}{B_{h}}\right)\;\times \;H\right]}$$

where *m*(*T*) is the rate of mortality at temperature *T*(°C). *T*_opt_ is the optimum temperature for survival (°C). *B*,* B*_l,_ *B*_h_, and *H* are the fitted parameters.

As rule of thumb, all the listed functions were chosen based on the lower value of their value.

#### 2.3.5 Longevity and fecundity

The longevity of *Brevicoryne brassicae* was determined using the mean survival time of an adult. The inversion of the mean longevity time allows us to calculate the senescence rate. The Stinner 4 model (Stinner, Gutierrez, & Butler, 1974) which had the lower AIC value was fitted to determine the relationship between the senescence rate and temperature. The mathematical expression of the model is illustrated as:

$$S\left(T\right)\;=\;\frac{C_{1}}{1\;+\;\exp(K_{1}\;+\;K_{2}T)}\;+\;\frac{C_{2}}{1\;+\;\exp(K_{1}\;+\;K_{2}\left(2T_{0}\;-\;T\right))}$$

*S*(*T*) is the senescence rate at temperature *T*(°C). *T*_0_ is the optimum temperature (°C). *C*_1_ and *C*_2_ are the maximum and minimum temperatures (°C) when *T* ≤*T*_o_ and *T* > *T*_o_, respectively. *K*_1_ and *K*_2_ are constants representing the slope and the intercept, respectively.

The fecundity was modeled by considering a total number of nymph produced per adult during the entire lifespan. The polynomial 2 function shown below was fitted to represent the temperature effect on this parameter.

$$f\left(T\right)\;=\;\exp\left(a\;+\;bT\;+\;cT^{2}\right)$$

#### 2.3.6 Simulation of life table parameters at constant temperatures

Life table parameters of *Brevicoryne brassicae* including gross reproductive rate (GRR), net reproductive rate (*R*_o_), intrinsic rate of natural increase (*r*_m_), finite rate of increase (ƛ), mean generation time (*T*), and doubling time (*D*_t_) were estimated using the module “stochastic simulation tool” in ILCYM, which is based on rate summation and cohort up‐dating approach (Curry et al., 1978). A female ratio of 1 was established for all the temperatures that were studied because of the parthenogenetic status of *B. brassicae*. For estimation of life table parameters, five replicates were performed with six simulations for each experimental temperature. The estimated life table parameters were plotted against respective temperatures and fitted to a polynomial function represented by this equation:

$$L_{P}\left(T\right)\;=\;a\;+\;bT\;+\;cT^{2}$$

where *L*_p_ (*T*) represents the respective life table parameters (GRR, *R*_0_, *T*,* r*_m_, l, *D*_t_) at temperature *T* (°C), and *a*,* b*, and *c* are the model parameters.

#### 2.3.7 Complementary analysis

Data on the development time of the different life stages, adult longevity, and fecundity were compared across constant temperatures using a one‐way ANOVA using the R software (version 3.5.1; R Development R Core Team, 2018) using the package called “agricolae” (de Mendiburu, 2017). When significant differences were detected, the Student–Newman–Keuls (SNK) post hoc test was used to separate the means (*p* < 0.05).

## 3 RESULTS

### 3.1 The relationship between constant temperature and the development of immature stage

**Figure 2:**
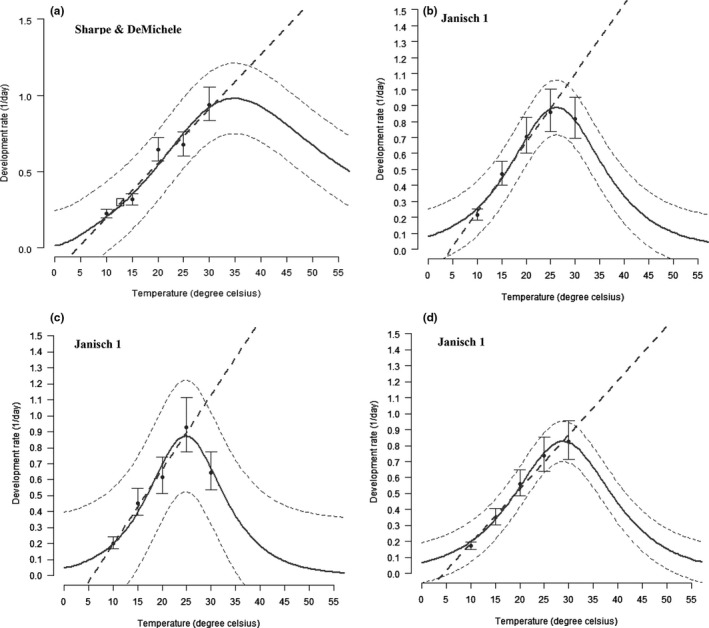
The temperature‐dependent developmental rate for immature stages of *Brevicoryne brassicae*. Nymph I (a), nymph II (b), nymph III (c), and nymph IV (d). The bold solid line is the selected model output, and dashed lines above and below represent the upper and lower 95% confidence bands. Bars represent the standard deviation of the mean

Developmental times of *Brevicoryne brassicae* immature stages significantly decreased with the increase in temperature, ranging from 18.45 days at 10°C to 5.05 days at 30°C (*F*_4‐461_ = 236.2; *p *<* *0.0001). No development occurred at 35°C for nymph I (Table 1). The temperature effect on development times of all nymphal stages was described by a cumulative logit distribution (Table 2). The estimated lower threshold temperatures were 1.64°C for nymph I, 1.57°C for nymph II, 1.56°C for nymph III, and 1.62°C for nymph IV. The thermal constants for nymphs I, II, III, and IV were estimated to be 0.88, 0.88, 0.08, and 0.79 DD, respectively (Table 3). The Sharpe & DeMichele 1 and Janisch 1 models were preferred to describe the relationship between temperatures and development rate of *B. brassicae* (Table 3 and Figure 2).

**Table 1:** Mean development time of *Brevicoryne brassicae* of nymphal stages at different constant temperatures in the laboratory

Temperature (^0^C)	Nymph I	Nymph II	Nymph III	Nymph IV	Nymph I to adults
10	4.5 ± 0.11a	4.65 ± 0.147a	4.974 ± 0.144a	5.834 ± 0.281a	18.45 ± 0.04a
15	3.2 ± 0.90b	2.12 ± 0.076b	2.204 ± 0.075b	2.852 ± 0.085b	10.37 ± 0.26b
20	1.6 ± 0.05c	1.418 ± 0.045c	1.619 ± 0.049c	1.78 ± 0.059 c	6.42 ± 0.07c
25	1.48 ± 0.03c	1.162 ± 0.032d	1.078 ± 0.038d	1.356 ± 0.035d	5.076 ± 0.09d
30	1.06 ± 0.00d	1.227 ± 0.033d	1.554 ± 0.066c	1.210 ± 0.000d	5.05 ± 0.10d
35		–	–	–	–

Similar letters (a, b, c, d) in the column indicate no significant differences (*p* < 0.05) at various constant temperatures by SNK test.

**Table 2:** Estimated parameters (mean ± *SE*) of the cumulative distribution functions fitted to normalized development time frequencies for immature life stages of *Brevicoryne brassicae*. Fitted functions: probit model (nymphs I, II, III, and IV)

Temperatures	Intercepts(ai)						Slope	AIC
	10°C	15°C	20°C	25°C	30°C	35°C		
Nymph I	−10.12 (0.46)	−7.77 (0.38)	−2.99 (0.28)	−2.65 (0.27)	−0.43 (0.20)	21.31 (2573.4)	6.75 (0.30)	105.50
Nymph II	−7.81 (0.34)	−3.82 (0.23)	−1.77 (0.21)	−0.76 (0.19)	−1.04 (0.20)	20.31 (1560.8)	5.08 (0.20)	192.68
Nymph III	−6.96 (0.33)	−3.43 (0.22)	−2.09 (0.20)	−0.33 (0.18)	−1.91 (0.19)	19.31 (946.71)	4.34 (0.19)	270.98
Nymph IV	−9.77 (0.46)	−5.81 (0.31)	−3.20 (0.25)	−1.69 (0.22)	−1.06 (0.2)	19.31 (946.71)	5.54 (0.25)	280.30

The number in parentheses represents standard errors.

**Table 3:** Estimated parameters (mean ± *SE*) of the nonlinear models (Sharpe & DeMichele, and Janisch 1) and linear model fitted to the temperature‐dependent development rate of immature life stages of *Brevicoryne brassicae*

Sharpe & DeMichele model									
Life stage		*H*_a_	*H*_h_	*H*_l_	*T*_0_	*T*_h_	Tl	AIC	
Nymph I	0.30	16785.11 (10820.872)	27315.33 (273.53)	−64209.01 (1.54)	285.8253	304.1645 (646.61)	276.64 (26.3)	7.0070	

	Janisch 1 model				Linear model				
	*D*_min_	*T*_opt_	*K*	AIC	Life stage	Intercept (*a*)	Slope (*b*)	*K*	*T*_min_ (°C)
Nymph II	1.13 (0.0)	26.18 (0.66)	0.11 (0.0)	−14.61	Nymph I	1.88 (0.06)	−1.14 (0.06)	0.88	1.65
Nymph III	1.14 (0.0)	24.81 (0.86)	0.15 (0.02)	−7.49	Nymph II	1.8 (0.07)	−1.15 (0.057)	0.87	1.57
Nymph IV	1.21 (0.0)	28.85 (0.95)	−0.11 (0.01)	−17.66	Nymph III	1.95 (0.07)	−1.25 (0.056)	0.80	1.56
					Nymph IV	2.06 (0.06)	−1.27 (0.05)	0.79	1.62

Numbers in parentheses are standard errors. *T*_min_ represents lower developmental threshold, calculated by intercept/slope, removing negative sign.

### 3.2 Mortality of nymphal stages

**Figure 3:**
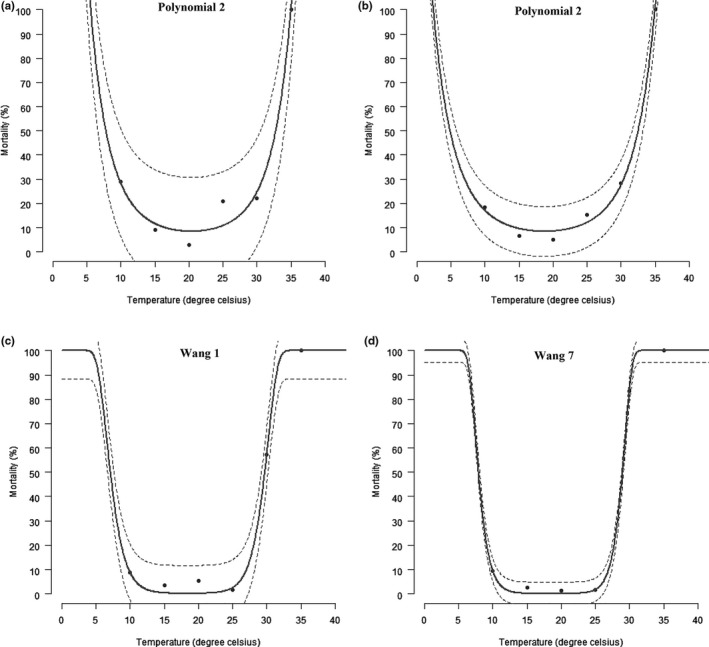
Temperature‐dependent mortality rates of immature *Brevicoryne brassicae* life stage. Nymph I (a), nymph II (b), nymph III (c), and nymph IV (d). The upper and lower 95% confidence intervals of the model are indicated. Markers are observed means

The mortality of *Brevicoryne brassicae* nymphal stages varied significantly among the rearing temperatures (Table 4). Mortality was highest at temperatures of 10, 30, and 35°C and lowest at temperatures of 15, 20, and 25°C for all immature stages. Accordingly, the revealed temperature effect in the mortality of *B. brassicae* was well visualized by the polynomial 2 function for nymphs I and II, Wang 1 function for nymph III, and Wang 7 function for nymph IV (Table 5 and Figure 3).

**Table 4:** Mean mortality of *Brevicoryne brassicae* life stages at different constant temperatures in the laboratory

Temperature (°C)	Mortality rate (% ± *SE*)			
	Nymph I	Nymph II	Nymph III	Nymph IV
10	29 ± 4.53a	18.31 ± 3.51a	8.62 ± 2.67a	9.43 ± 2.7a
15	9 ± 2.86b	6.59 ± 2.46b	3.53 ± 1.83b	2.44 ± 1.54b
20	3 ± 1.70c	5.15 ± 2.20b	5.34 ± 2.23b	1.15 ± 1.05b
25	21 ± 4.07d	15.19 ± 3.4a	1.5 ± 1.2c	1.51 ± 1.21b
30	22 ± 4.1d	28.20 ± 3.46c	57.14 ± 2.03d	83.33 ± 0.46c
35	100 ± 10e	–	–	–

Within a column, means followed by the same letters are not significantly different (*p* < 0.05), Student–Newman–Keuls test.

**Table 5:** Estimated parameters (mean ± *SE*) of the nonlinear models fitted to mortality rate for immature life stages of *Brevicoryne brassicae*: Polynomial 2 (nymphs I and II), Wang 1(nymph III), and Wang 7 (nymph IV)

Life stage	Polynomial 2 model							
	Intercept (*a*)	*B*	*C*	AIC	*F*	*df*	*p*	
Nymph I	2.24 (0.003)	−0.46 (0.02)	0.01 (0.00)	−11.03	61.78	(2,3)	0.0036	
Nymph II	0.78 (0.001)	−0.35 (0.01)	0.01 (0.00)	−20.32	306.17	(2,3)	0.0003	

	Wang 1 model							
	*T*_opt_	*B*	*H*	AIC	*F*	*df*	*p*	
Nymph III	18.42 (0.00)	1.42 (0.00)	0.0002 (0.00)	−18.93	310.13	(2,3)	0.0003	

	Wang 7 model							
	*T*_opt_	Bl	Bh	*H*	AIC	*F*	*df*	*p*
Nymph IV	28.14 (0.26)	126.96 (32.81)	1.09 (0.13)	7.88 (0.23)	−28.87	1298.93	(2,3)	0.0004

The numbers in parentheses are standard errors.

### 3.3 Longevity and fecundity capacity of adult

**Figure 4:**
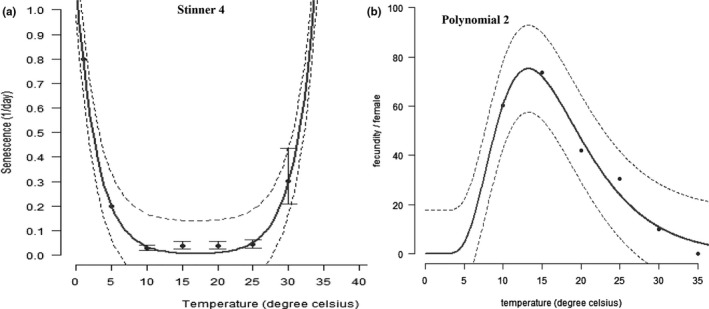
Temperature‐dependent longevity curve (a) and total nymph produced per adult curve (b) of *Brevicoryne brassicae*. The upper and lower 95% confidence intervals of the model are indicated. Markers are observed means; bars represent standard deviation

Temperature had a significant effect on the longevity (*F*_4‐277_ = 10.8; *p *<* *0.0001) and fecundity (*F*_3‐274_ = 23.19; *p *<* *0.0001) of *Brevicoryne brassicae* (Table 6). The longest longevity period was recorded at 10°C and the shortest at 30°C (Table 6). The relationship between temperature and mean senescence rate of *B. brassicae* was represented by Stinner 4 function (Table 7 and Figure 4a). Our results showed that optimal fecundity was obtained from temperatures ranging between 10 and 20°C. The effect of temperature on the fecundity of *B. brassicae* was well described by the polynomial 2 function (Table 7 and Figure 4b).

**Table 6:** Mean longevity and fecundity at rearing temperatures

Temperature (°C)	Longevity (days ± *SE*)	Fecundity (nymphs/female)
10	35.07 ± 1.38a	41.38 ± 3.04a
15	25.66 ± 1.32b	39.84 ± 1.98a
20	25.9 ± 1.15b	46.36 ± 1.73a
25	23.21 ± 0.95b	23.57 ± 1.46b
30	3.32 ± 0.25c	0
35	No adults	0

Within a column, means followed by the same letters are not significantly different (*p* < 0.05), Student–Newman–Keuls test.

**Table 7:** Estimated parameters (mean ± *SE*) of Stinner 4 and Polynomial 2 models fitted to senescence and fecundity for adults of *Brevicoryne brassicae*

	Senescence								
Stinner 4 model	*C*_1_	*C*_2_	*K*_1_	*K*_2_	*T*_0_	AIC	*df*	*F*	*p*
	16.31 (0.15)	14.87 (4.89)	2.61 (0.003)	0.35 (9.85)	16.79 (0.78)	−25.44	(4,2)	121.55	0.0082

	Fecundity								
Polynomial 2 model	*A*	*B*	*c*	AIC	*df*	*F*	*p*		
	0.61 (1.21)	0.39 (0.15)	−0.01 (0.004)	55.40	(2,4)	13.31	0.017		

The numbers in parentheses are the standard error.

### 3.4 Demographic parameters of *Brevicoryne brassicae* at constant temperatures

**Figure 5:**
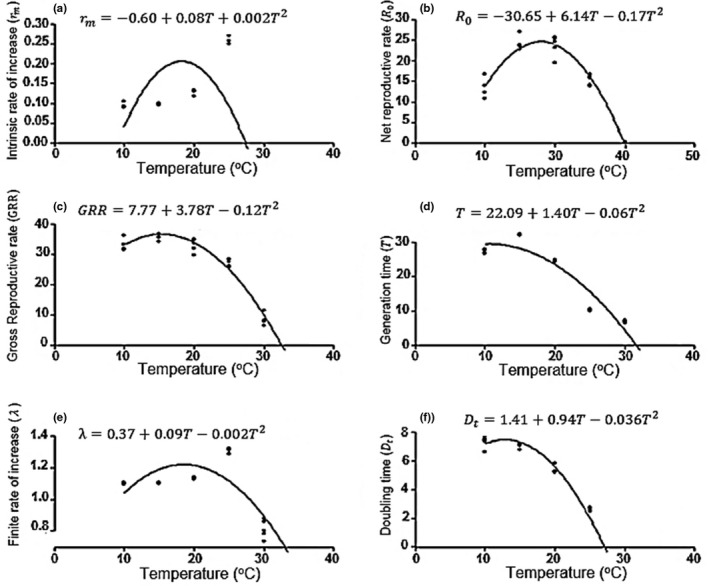
Life table parameters of *Brevicoryne brassicae* estimated through model prediction among a tested temperature: (a) Intrinsic rate of natural increase (*r*_m_); (b) net reproduction rate (*R*_o_); (c) gross reproductive rate (GRR); (d) mean generation time (*T*); (e) finite rate of increase (ƛ); and (f) doubling time (*D*_t_)

Results presented in Table 8 show that in *Brevicoryne brassicae*, the intrinsic rate of increase (*r*_m_), the net reproduction rate (*R*_0_), the mean generation time (*T*), the finite rate of increase (λ), the doubling time (*D*_t_), and the gross reproduction rate (GRR) were significantly influenced by the temperature (*r*_m_: *F*_4‐166_ = 1092.86, *p *<* *0.0001; *R*_o_: *F*_4‐166_ = 1112.42, *p *<* *0,0001; *T*:* F*_4‐166_ = 24704.4, *p *<* *0.0001; λ: *F*_4‐166_ = 1806.78, *p *<* *0,0001; *D*_t_: *F*_4‐166_ = 4155.05, *p *<* *0,0001; and GRR: *F*_4‐166_ *= *1386.32, *p *<* *0,000). *r*_m_ was highest at 25°C (0.224 ± 0.007), and lowest at 30°C (−0.33 ± 0.014). *R*_0_ varied from 10.23 ± 0.21 to 0.15 ± 0.01 females/female/generation at temperatures of 10 and 30°C, respectively. *T* varied from 36.85 ± 1.5 days (15°C) to 6.86 ± 0.1 days (30°C). λ was maximal at 25°C (1.22 ± 0.036). *D*_t_ decreased with the increase in temperature, whether 8.32 ± 0.1 days (10°C) and 2.2 ± 0.1 days (30°C). GRR was highest at temperatures of 15 (3.08 ± 1.5) and 20°C (33.42 ± 1.05). The polynomial model showed that temperatures between 15°C and 25°C are adequate for an increase in *B. brassicae* population (Figure 5).

**Table 8:** Life table parameters (mean ± *SE*) of *Brevicoryne brassicae* at different constant temperatures. Intrinsic rate of increase (*r*_m_), net reproduction rate (*R*_o_), mean generation time (*T*), finite rate of increase (λ), doubling time (*D*_t_), gross reproduction rate (GRR)

Temperature(°C)	Parameters					
	*r*_m_	*R*_o_	*T*	λ	*D*_t_	GRR
10	0.0836 ± 85 × 10^−5^a	10.23 ± 0.21a	27.73 ± 0.11a	1.08 ± 0.001a	8.32 ± 0.1a	27.31 ± 0.3a
15	0.080 ± 0.003a	22.22 ± 1.02b	36.85 ± 1.5b	1.12 ± 0.043b	8.10 ± 0.35b	33.08 ± 1.5b
20	0.11 ± 0.003b	24.04 ± 0.8c	26.89 ± 0.8a	1.09 ± 0.032b	5.83 ± 0,18c	33.42 ± 1.05b
25	0.224 ± 0.007c	11.06 ± 0.3d	10.21 ± 0.3c	1.22 ± 0.036c	2.92 ± 0.08d	18.96 ± 0.6c
30	−0.33 ± 0.014d	0.15 ± 0.01e	6.86 ± 0.1d	0.72 ± 0.01d	−2.2 ± 0.1e	3.64 ± 0.28d
35	–	–	–	–		–

Within a column, means followed by the same letters are not significantly different (*p *< 0.05), Student–Newman–Keuls test.

## 4 DISCUSSION

The results of this research provided reaction norms that depict the effect of temperature on the biological parameters of *Brevicoryne brassicae*. This was performed using a friendly‐user software called Insect Life Cycle Modelling (ICLYM), in which the aim is to assist researchers in developing insect temperature‐based models (Tonnang et al., 2013). The choice of appropriate models that describe the biological response of an insect to different temperatures should be based on their unimodal shape that predicts the lower, the optimal, and the upper thermal requirement of the insect (Mirhossein et al., ; Régnière, Powell, Bentz, & Nealis, 2012). In the present study, all the models selected accommodated the precedent curvilinear relationship.

*Brevicoryne Brassicae* successfully developed from 10°C to 30°C with the decrease in developmental time. However, no development occurred 35°C. This could be attributed to the effect of temperature on the metabolic activity of *B. brassicae*. Indeed, temperature is a crucial factor that influences the development of insects (Angilletta, Steury, & Sears., 2004; Brown, Gillooly, Allen, Savage, & West, 2004; Porter, Parry, & Carter, 1991). The functions Sharpe & DeMichele and Janisch used in illustrating the relationship between temperature and the developmental rate of *B. brassicae* predicted that their optimal developmental temperatures are comprised between 20 and 30°C. The same models showed that temperatures below 20°C and above 30°C retarded the development of this aphid. This prediction is in line with other works in which mathematical functions were not included. For instance, Fathipour et al. (2005) and Satar et al. (2005) showed that *B. brassicae* optimally developed at 20°C and the alternating temperature of 25/30°C, respectively. Additionally, Abdel‐Rahman, Awad, Omar, and Mahmoud (2011) reported that 28°C was the optimal developmental time of *B. brassicae*. The functions Sharpe & DeMichele and Janisch used in our study also successfully predicted temperature range of development in *Phenacoccus solenopsis* (Fand et al., 2014) and *Liriomyza huidobrensis* (Mujica, Sporleder, Carhuapoma, & Kroschel, 2017), two insect species with a wide range of thermal tolerance and cosmopolitan distribution as *B. brassicae*.

The linear equation used in this study predicted 1.64 C, 1.59 C, 1.56 C, and 1.62°C were the lower lethal temperatures for nymph I, nymph II, nymph III, and nymph IV, respectively. This could explain the presence of *B. brassicae* in the temperate zones where temperatures decrease dramatically in winter (Hines & Hutchison, 2013). Our prediction corroborates the result of Markkula (1953) who found that 1.7°C was the lower lethal temperature of *B. brassicae* immature. This indicates that the linear equation used in this study accurately predicted the lower temperature threshold of *B. brassicae*. The linear equation has been extensively used other works to determine the lower temperature thresholds of different insect species (see: Azrag, Murungi, Tonnang, Mwenda, & Babin, 2017; Fand, Tonnang, Kumar, Kamble, & Bal, 2014; Tanga et al., 2018; Tazerouni & Talebi, 2014).

The mortality rate of the different nymphal stages of *Brevicoryne brassicae* was highest at extreme temperatures (10°C, 30°C and 35°C) and low at 15°C, 20°C, and 25°C. The polynomial 2, Wang 1, and Wang 7 functions used in this study clearly illustrated the obtained. Smith (1999) explained that nymphs of *B. brassicae*, in comparison with adults, have a small size and a thin layer of wax coating (Figure 1), which play a useful role in the protection of aphids against freezing at low temperatures and desiccation at high temperatures. In younger stages of *Phenacoccus solenopsis* which also possess a thin protective wax coating, Fand et al. (2014), using polynomial and Wang functions, also predicted higher mortality at the temperatures below 10°C and above 30°C.

The longevity and fecundity of adults of *B. brassicae* were also affected by temperature. They were maximal respectively at 10 and 25°C. The Stinner function fitted well with our longevity data. This model illustrates that adults of *B. brassicae* have an optimal lifespan between 10°C and 25°C. We recorded the highest longevity of *B. brassicae* at 10°C (35.07 days), while Satar et al. (2005) found the highest longevity at 15°C (16.3 days). This difference could be ascribed to the dissemblance in our respective methodology, or the strains of *B. brassicae* used. However, the precedent temperatures (10 and 15°C) occur within the optimal longevity temperature range predicted by our model, illustrating therefore, the performance of the Stinner model in predicting temperature effect in *B. brassicae* longevity. Fletcher, Axtell, and Stinner (1990) used the same function to model the effect of temperature on the longevity of the house fly (*Musca domestica*). The polynomial function we used to model the effect of temperature on *B. brassicae* fecundity predicted that temperatures between 10 and 30°C are more favor *B. brassicae* fecundity. We obtained the highest fecundity value at 20°C (46.36 nymphs). However, this value was low compared to the value obtained by Akinlosotu (1977) at the same temperature (74.5 nymphs). Also, Satar et al. (2005) reported the highest fecundity per day at 25°C (4.2 nymphs). The difference observed in our respective results could be attributed to the different type of cabbage cultivars used in our respective experimentations. Akinlosotu (1977) used the Gemmifera cultivar, while Satar et al. (2005) used the Capitata cultivar. However, in our study, we used the Marcanta cultivar. Indeed, the literature has demonstrated the effect of cabbage cultivar in *B. brassicae* fecundity (Ellis & Farrell, 1995; Gia & Andrew, 2015; Maremela, Tiroesele, Obopile, & Tshegofatso, 2013). Despite that difference, 20 and 25°C still represent the temperatures that favour reproduction in *B. brassicae*, as predicted by our selected model. This further demonstrates the efficacy of the polynomial function in modeling the effect of temperature on *B. brassicae* fecundity. The polynomial function has been highlighted as one of the best models for modeling the temperature effect on reproduction of several insect species including *Phthorimaea operculella* (Sporleder et al., 2004), *Chilo partellus* (Khadioli et al., 2014), *Antestiopsis thunbergii* (Azrag et al., 2017), and *Symmetrischema tangolias* (Sporleder, Schaub, Aldana, & Kroschel, 2017).

The life table parameters of *B. brassicae* obtained based on the stochastic simulation significantly differed among the treatment temperatures. The net reproductive rate (*R*_0_) and the gross reproductive rate (GRR) were high at 20°C compared with the other temperatures. This high value at 20°C was resultant of the low mortality of *B. brassicae* nymphal stages at that temperature. The lowest generation time (*T*) occurred at 25°C (10.21 days) and 30°C (6.86 days) due to the rapid development of nymph occurred in these temperatures. This indicates that, during the maturation period of cabbage (between 2.5 and 3.5 months depending to the cultivar), one plant can host at least nine generations of *B. brassicae*, justifying, therefore, the severe losses that occur *B. brassicae* infestation (70%–80%). The intrinsic rate of increase (*r*_m_) and the finite population rate (λ) were maximal at 25°C and minimal at 30°C. The highest *r*_m_ value obtained 25°C is justified by the rapid development and reproduction that occurred at that temperature, while the negative *r*_m_ value obtained at 30°C was due to the high mortality of the nymphs and the absence of fecundity at that temperature. Kocourek, Havelka, Berankova, and Jarosik (1994) and Southwood and Henderson (2000) previously explained that several biotic factors such as fertility, survival, and generation time affect the intrinsic rate of increase, thus rendering this parameter more adequate in describing the physiological qualities of insects. Our stochastically simulated *r*_m_ value (0.224) obtained at 25°C is largely consistent with earlier reports. For instance, Hoseini, Fathipour, and Talebi (2003), Fathipour et al. (2005), and Gupta (2014) obtained an *r*_m_ value of 0.25, 0.22, and 0.252 at 25°C, respectively. The statement of this reports further validates the accuracy of the temperature‐dependent models used in predicting the response of *B. brassicae* to the temperature effects in this current study.

In general, this study provides mathematical functions that describe the effect of temperature on several biological parameters of the cabbage aphid *Brevicoryne brassicae*. Our models show that the population of *B. brassicae* can be easily found in environments where temperatures fluctuate between 15 and 30°C. Subsequent studies in which the biological parameters of *B. brassicae* are recorded under fluctuating temperatures are requested. These would help to validate the developed temperature‐based models used in this study and further help to map the likely changes in the distribution and abundance of *B. brassicae* in response to global warming. This information will be useful for implementing management programs against *B. brassicae* in cabbage fields.

## CONFLICT OF INTEREST

None declared.

## AUTHOR CONTRIBUTIONS

SBBS designed the study, collected and analyzed the data, and wrote the manuscript. NNS and DM assisted in data analysis and interpretation. KS and DM proofread the manuscript.

## DATA ACCESSIBILITY

The authors confirm that all the data supporting the results of this manuscript will be immediately archived in the Dryad system upon acceptance.
