# Corrigendum

**DOI:** 10.1002/ece3.5018

**Published:** 2019-03-07

In “Effect of temperature on the biological parameters of the cabbage aphid *Brevicoryne brassicae,*” which was published in issue December 23 2018, the name of one of the authors, the Dryad link and the author contribution sections were incorrect. The correct information is printed below.

- Hannah Rachid name should be Rachid Hanna
- Author Contributions section should be: The research in this paper is part of the senior author’s MSc thesis at the University of Yaoundé I which was supervised by Drs R. Hanna and S. Kekeunou. This study was supported by the International Institute of Tropical Agriculture with funds from German Federal Ministry for Economic Cooperation and Development (BMZ) with R. Hanna as project leader. We would like to thank all the colleagues including Abdelmutalab Ahmed, Caroline Kungu, Ritter Guimapi, and Olabimpe Olaide who generously provided advice, information, and suggestions to improve the quality of this manuscript.
- Dryad link is https://doi.org/10.5061/dryad.6sf5184.
